# Sulfonated Cellulose: A Strategy for Effective Methylene Blue Sequestration

**DOI:** 10.1021/acsomega.5c00179

**Published:** 2025-02-21

**Authors:** Mustafa Toy, Yaşar Kemal Recepoğlu, Özgür Arar

**Affiliations:** † Department of Chemistry, Faculty of Science, Ege University, Bornova, Izmir 35040, Türkiye; ‡ Department of Chemical Engineering, Faculty of Engineering, Izmir Institute of Technology, Urla, Izmir 35430, Türkiye; § Department of Chemistry, Faculty of Science, Ege University, Bornova, Izmir 35040, Türkiye

## Abstract

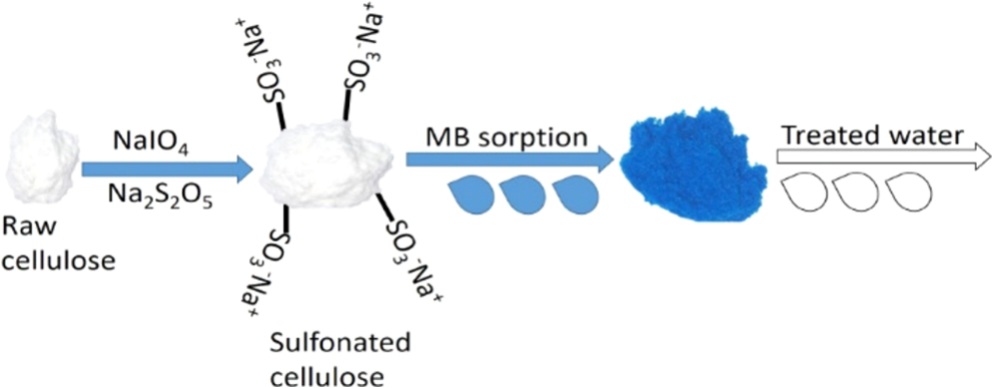

This study investigates the sulfonation modification of cellulose for the removal of methylene blue (MB) from aqueous solutions. The prepared biosorbent was characterized, and its sorption capacity, kinetics, and thermodynamics were systematically evaluated. Fourier-transform infrared (FTIR) spectroscopy analyzed structural modifications, while scanning electron microscopy (SEM) examined the surface properties. The optimal sorbent dosage was determined as 0.05 g. MB removal efficiency increased from 11% at pH 1 to 70% at pH 2, reaching 99% within the pH range of 3 to 7. Kinetic studies revealed rapid sorption, achieving 99% removal within 3 min. Among various isotherm models, the Langmuir model provided the best fit (*R*^2^ = 0.9989), indicating monolayer sorption with a maximum capacity of 37.65 mg/g. Thermodynamic analysis showed negative Δ*G*° values, confirming a spontaneous sorption process, while an enthalpy change (Δ*H*°) of −33.5 kJ/mol indicated exothermic behavior. The entropy change (Δ*S*°) of −82.6 J mol^–1^·K^–1^ suggested decreased disorder during sorption. Regeneration studies demonstrated that 0.2 M HCl combined with ethanol achieved the highest desorption efficiency, and after three cycles, the MB removal efficiency remained above 99%. The presence of −SO_3_^–^ groups played a crucial role in MB sorption via ion exchange and may also contribute through hydrogen bonding, thereby enhancing MB sorption. These findings highlight sulfonated cellulose as an efficient and regenerable biosorbent for MB removal, offering valuable insights into its sorption mechanisms.

## Introduction

The adverse effects stemming from the contamination of water resources restrict the demand for clean water and lead to detrimental impacts on the aquatic ecosystem. The pollution of existing water sources will result in significant challenges and problems in the future.^1−3^ Chemical substances that facilitate our daily lives but also give rise to harmful environmental effects through their widespread use can be broadly categorized into three groups: detergents, pharmaceuticals, and pigments. Pigments, found predominantly in the effluents of textiles, food, cosmetics, paper, and paint manufacturing industries, constitute significant pollutants classified into anionic, cationic, and nonionic dyes.^4−6^ Since a substantial portion of the organic structure in wastewater and dye groups includes aromatic rings, these compounds contain carcinogenic substances, posing a severe threat to the environment and ecosystems. Consequently, ongoing efforts are being made to minimize the degree of damage and level of risk through preventive measures.^7^ Conventional treatment methods, such as biological treatment and coagulation-flocculation, often need to be improved to purify wastewater containing dyestuffs. The treatment of dyed wastewater using conventional methods is highly challenging. The sorption method stands out as one of the most effective treatment techniques due to its simple design and cost-effectiveness in operation.^8^ The dye substance methylene blue (MB) is utilized in the dyeing processes of textiles, paper, leather, pulp factories, food, and plastics, but its release into water bodies poses significant environmental risks due to its high chemical stability, toxicity to aquatic organisms, and potential to bioaccumulate. Depending on its concentration in wastewater, prolonged exposure to MB can induce health problems in organisms, not only eye irritation, nausea, and vomiting but also respiratory and neurological disorders. It also leads to a slowing of light refraction in water bodies, disrupting photosynthesis and aquatic ecosystems and, hence, an increase in chemical and biological oxygen demand.^9^ To remove MB from water, various treatment processes have been applied, including photocatalytic degradation,^10−13^ sonochemical degradation,^14,15^ surfactant-enhanced ultrafiltration,^16,17^ cation-exchange membranes,^18−20^ electrochemical degradation,^21−26^ integrated chemical-biological degradation,^27,28^ and adsorption.^29−35^ Since synthetic dyes in wastewater cannot be efficiently decolorized using conventional methods, sorption onto inexpensive and efficient solid supports is considered a simple and economical method for their removal from water and wastewater.^36,37^ For instance, wheat shells were utilized for the removal of MB from aqueous solutions, and maximum adsorption capacity was calculated at different temperatures (303, 313, and 323 K), 16.56, 20.83, and 21.50 mg/g, respectively, where equilibrium was attained within 60 min.^38^ Furthermore, the development of novel adsorbents based on cellulose is pivotal in addressing contemporary environmental challenges while promoting sustainability. The abundance, renewability, and biodegradability of cellulose make it an ideal foundation for eco-friendly adsorbent materials. Through versatile modifications, cellulose-based adsorbents can be tailored to enhance adsorption capacity, selectivity, and other desirable properties. The high surface area and porosity of cellulose, along with its compatibility with other materials, further underscore its significance in advancing efficient and versatile adsorption technologies.^39,40^ The inherent limitations of native cellulose, such as limited adsorption capacity and selectivity, are overcome through a diverse range of modifications.^41^ Chemical modifications, including esterification,^42^ etherification,^43^ and acetylation,^44^ modify hydrophobicity and polarity of cellulose, tailoring its surface properties for improved adsorption of specific contaminants.^45−47^ Physical modifications, such as cross-linking and nanostructure formation, contribute to increased mechanical strength and a higher surface area, enhancing accessibility to active adsorption sites.^48,49^ Ionic modifications, achieved through sulfonation or phosphorylation, impart ionic properties to cellulose, rendering it effective for sorbing heavy metals and ions.^50−52^ Biological modifications, including enzymatic treatments, offer a sustainable approach to altering cellulose surface characteristics.^53,54^ The choice of a specific modification strategy depends on the targeted pollutants and the desired attributes of the cellulose-based adsorbent for optimal adsorption performance.

In this study, cellulose was modified through sulfonation to remove MB from aqueous solutions, as sulfonated cellulose is to be claimed as an eco-friendly and cost-effective biosorbent with high adsorption efficiency for MB removal. The introduction of sulfonic acid groups enhances its affinity for cationic dyes through electrostatic interactions, improving the sorption capacity. Its renewable nature, chemical stability, and potential for regeneration make it a sustainable alternative to conventional adsorbents, offering an efficient solution for wastewater treatment. The primary objectives include the characterization of the prepared biosorbent and the assessment of the sorption capacity, kinetics, and thermodynamics of the process. The study aims to explore the influence of various parameters, such as sorbent dosage, initial dye concentration, contact time, and temperature, on sorption efficiency. Additionally, the research seeks to elucidate the underlying mechanisms governing the interaction between MB and sulfonated cellulose and the desorption/regeneration efficiency. This study provides pioneering insights into the application of sulfonated cellulose as a high-performance biosorbent for dye removal, advancing the fundamental understanding of its sorption mechanisms. The findings not only enhance environmental remediation strategies but also play a crucial role in optimizing sustainable water treatment technologies, offering a novel and eco-friendly approach to pollutant removal.

## Results and Discussion

### Characterization of Prepared Biosorbent

#### FTIR Analysis

**Figure 1:**
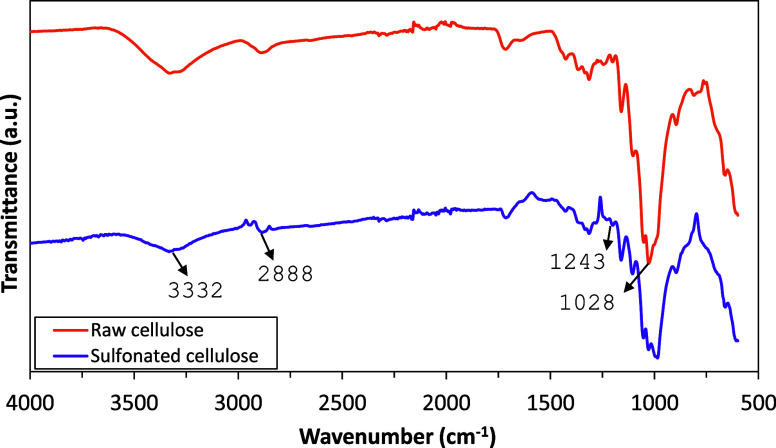
Comparative FTIR spectra of raw and sulfonated cellulose.

Figure 1 shows comparative Fourier-transform infrared (FTIR) spectra of raw and sulfonated cellulose. In the spectrum, characteristic peaks associated with cellulose functionalities are evident, such as the strong bands around 3332 cm^–1^ corresponding to stretching vibration frequencies of O–H, and this peak also includes inter- and intramolecular hydrogen bond vibrations in cellulose. The prominent peaks at 2900–2800 cm^–1^ are attributed to CH_2_ and C–H stretching vibrations. Moreover, the band at 1243 cm^–1^ should undoubtedly be attributed to internal deformation vibrations of the CH_2_ group. Upon sulfonation, notable changes are observed, particularly in the region between 1200 and 1000 cm^–1^, where new peaks may appear, indicative of the introduction of sulfonate groups. Additionally, alterations in the intensity and position of peaks associated with C–O and C–C stretching vibrations may signify changes in the cellulose backbone. The peak at 1028 cm^–1^ can be attributed to C–O stretching vibration in raw cellulose.^55−57^

#### SEM Analysis

**Figure 2:**
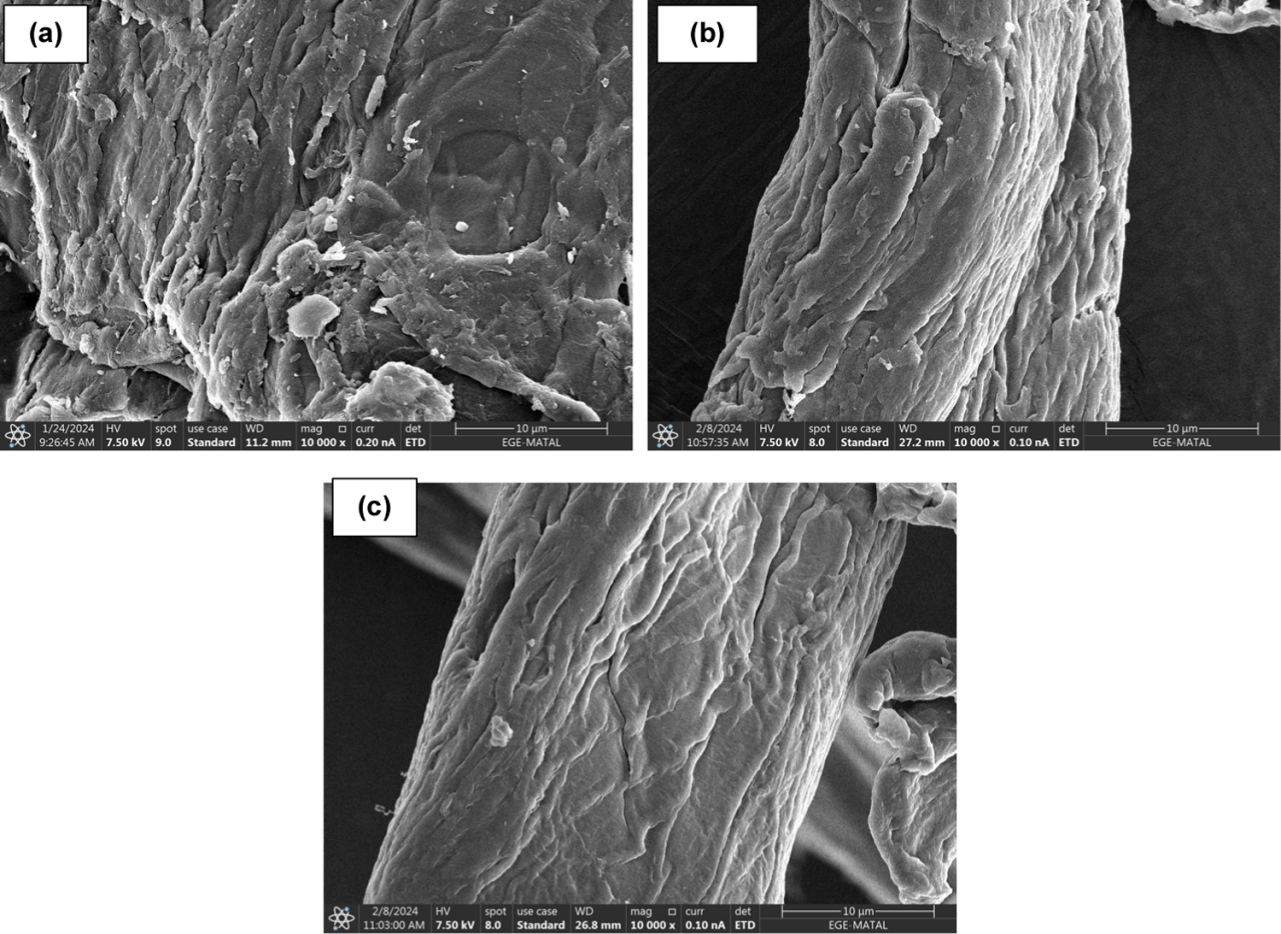
SEM images of (a) raw, (b) sulfonated, and (c) MB-loaded sulfonated cellulose at 10,000 times magnification.

Scanning electron microscopy (SEM) analysis provides valuable insights into the morphological characteristics of materials on a microscale level. Figure 2 illustrates SEM images of raw, sulfonated, and MB-loaded sulfonated cellulose at 10,000 times magnification. Distinct features emerge when SEM images are given among raw cellulose (Figure 2a), sulfonated cellulose (Figure 2b), and MB-loaded sulfonated cellulose (Figure 2c). Raw cellulose typically exhibits a fibrous and densely packed structure with well-defined cellulose fibers. Following the sulfonation process, the fibril structure of cellulose is effectively preserved, ensuring the material retains its intrinsic physical characteristics. This preservation is crucial for maintaining the integrity and functionality of the cellulose-based sorbent. Subsequent to the sorption of methylene blue (MB), analysis reveals that the sorbent’s structure remains unaltered, indicating that the fibril architecture is maintained even after the sorption process.

#### Elemental Analysis

In addition, elemental analysis using a LECO CHNS-932 analyzer revealed the elemental composition of the raw cellulose as 41.4% carbon (C) and 6.2% hydrogen (H). After sulfonation of the cellulose, the composition of the sorbent changed to 40.7% C, 6.0% H, and 0.2% sulfur (S). The presence of sulfur indicates that sulfonic acid groups were successfully attached to the sorbent. Furthermore, after methylene blue (MB) sorption, the composition of the sorbent changed to 42.6% C, 6.1% H, 0.5% nitrogen (N), and 0.3% S. The presence of nitrogen and the increase in sulfur content confirm that MB was adsorbed by the sorbent as MB contains both nitrogen and sulfur in its molecular structure.

### MB Removal Studies by Sulfonated Cellulose Biosorbent

#### Effect of Biosorbent Dose

**Figure 3:**
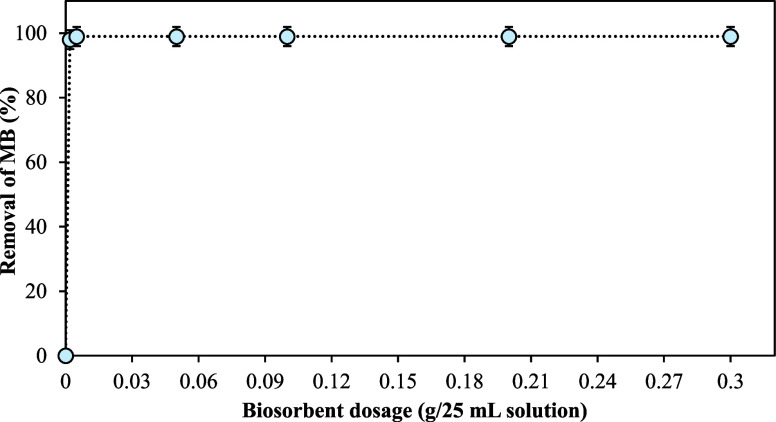
Effect of sulfonated cellulose biosorbent amount on the removal of MB from aqueous solution.

The effect of the sulfonated cellulose biosorbent dose plays a pivotal role in influencing dye removal efficiency from water. Figure 3 illustrates the effect of the sulfonated cellulose biosorbent dosage on the removal of methylene blue (MB) from an aqueous solution. When 0.002 g of the sorbent was applied, 98% of the MB dye was removed. Increasing the sorbent dose to 0.005 g further enhanced the removal efficiency to 99%. This improvement is attributed to the greater availability of functional groups for MB adsorption as the sorbent quantity increases. At lower dosages, the biosorbent may exhibit a limited number of active binding sites, restricting dye uptake and thereby reducing removal efficiency. Conversely, higher biosorbent dosages provide a greater number of accessible active sites, promoting enhanced MB sorption.^58^ However, precise weighing of 0.005 g of sorbent presents practical challenges; therefore, a dosage of 0.05 g was selected as the optimal sorbent quantity for subsequent parametric investigations.

#### Effect of pH on MB Removal

**Figure 4:**
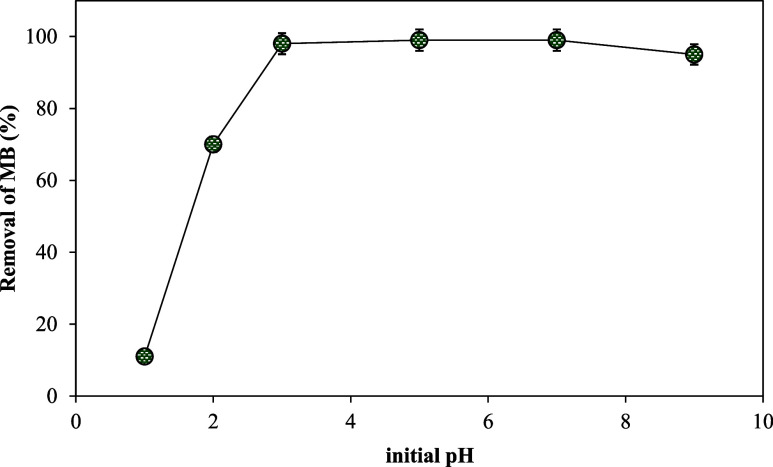
Effect of initial solution pH on MB removal by sulfonated cellulose.

The acidic or alkaline nature of the medium can influence the surface charge of the biosorbent and the speciation of the target pollutant. Systematic studies across a range of pH values are essential to elucidate the optimal conditions for maximizing the efficiency of MB removal in water treatment applications. The removal of MB using sulfonated cellulose biosorbent within the pH range of 1–9 is depicted in Figure 4. The interaction between the sulfonic acid groups and cations occurs through nonspecific electrostatic attraction. At pH 1 and 2, the high concentration of H^+^ ions, introduced to adjust the solution pH, significantly interferes with MB sorption. Since both H^+^ and MB cations carry the same positive charge, they compete for the sorption sites. However, due to its smaller size and higher concentration, H^+^ exhibits a stronger electrostatic interaction with sulfonic acid groups. Electrostatic interactions are inversely proportional to the hydrated radius, and MB, being a larger molecule with a greater hydrated radius, experiences weaker electrostatic attraction compared to that of H^+^. Consequently, sulfonic acid groups preferentially bind H^+^, reducing the removal efficiency of MB. As the pH increases to 3, the concentration of H^+^ in the solution decreases, reducing competition for active sites. This allows MB cations to interact more effectively with sulfonic acid groups, enhancing the sorption capacity and removal efficiency. Consequently, the optimum operational pH range was found to be between 3 and 7 with 99% MB removal by sulfonated cellulose as it started to decrease from 99 to 95% when the initial pH of the MB solution was 9. For practical applications, the pH range of 3–7 corresponds to typical conditions found in many industrial effluents and natural water sources. On the other hand, the pH stability within this range indicates that sulfonated cellulose can maintain its effectiveness under a wide range of water conditions, reducing the need for pH adjustments in treatment processes. On top of that, at extreme pH values (either acidic or basic), undesirable side reactions such as cellulose degradation or the formation of secondary pollutants may occur. Operating within the pH of 3–7 helps avoid these risks, ensuring the longevity and sustainability of sulfonated cellulose as a biosorbent.

#### Sorption Kinetics

**Figure 5:**
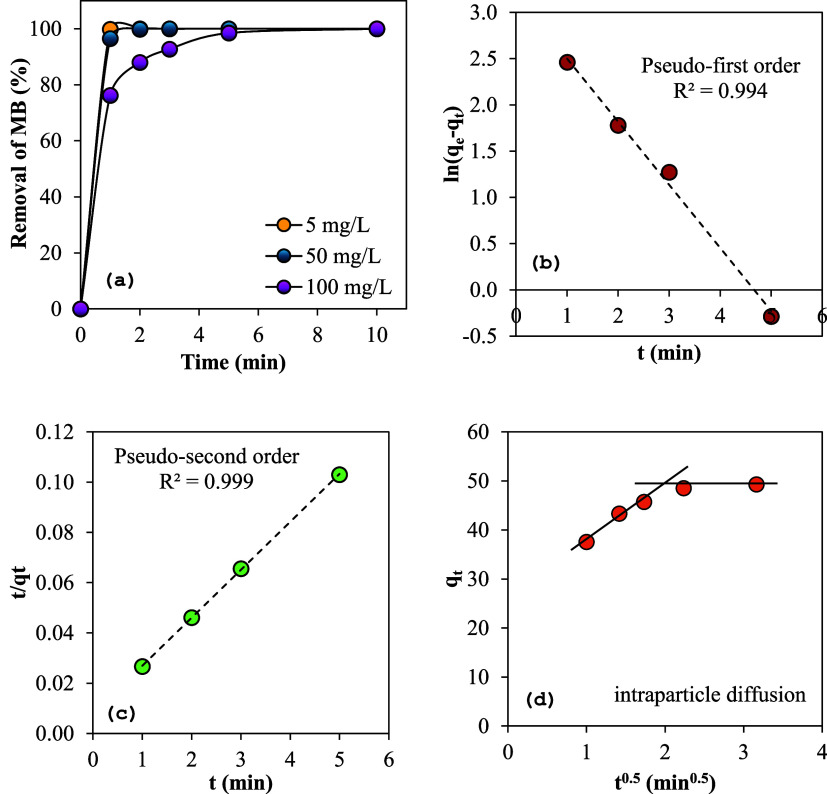
(a) Effect of contact time on MB removal efficiency for three different initial dye concentrations, (b) linear plot of pseudo-first-order model, (c) linear plot of pseudo-second-order model, and (d) intraparticle diffusion kinetics of MB sorption on sulfonated cellulose.

The efficiency of MB removal over time, as shown in Figure 5a, was determined from the analysis of the collected samples with three different initial MB concentrations. The prepared sorbent exhibited exceptionally rapid removal kinetics, reaching a 99% removal rate within just 3 min (for 5 and 50 mg/L). Cellulose-based biodegradable sorbents are recognized for their swift sorption kinetics, attributed to the functional groups present on their surface.^51,59−61^ Understanding kinetic parameters is essential for accurately predicting sorption rates, which provides important information for designing and modeling sorption processes. Consequently, three commonly used kinetic models were utilized to analyze the experimental data on the sorption of MB onto sulfonated cellulose: the Lagergren pseudo-first-order model (eq 1), the pseudo-second-order equation^62,63^ (eq 2), and the Weber and Morris intraparticle diffusion model (eq 3).^64^
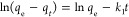
1
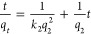
2
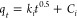
3where *q*_e_ (mg/g) and *q*_*t*_ (mg/g) are the amounts of dye sorbed at equilibrium and the amount of dye sorbed at time *t*, respectively. *t* is the time (min), *k*_1_ is the constant rate of pseudo-first-order adsorption (1/min), *k*_2_ is the constant rate of the pseudo-second-order adsorption (g/(mg·min)), *k*_*i*_ is the intraparticle diffusion rate (mg/(g·min^0.5^)), and *C*_*i*_ is the intercept.

Figure 5b,c illustrates the pseudo-first-order and pseudo-second-order models’ plots (for 100 mg/L), demonstrating the alignment between experimental data and the models using linear regression. By analyzing the slope and intercept of the ln(*q*_e_ – *q_t_*) vs *t* plot, the first-order rate constant (*k*_1_) and the calculated equilibrium sorption capacities (*q*_e,cal_) were derived. The correlation coefficient (*R*^2^) for MB sorption using the Lagergren equation was notably high at 0.994. However, the experimental equilibrium sorption capacity (*q*_e,exp_) of 49.30 mg/g did not align with the calculated *q*_e,cal_ value of 24.12 mg/g. This discrepancy indicates that MB sorption onto sulfonated cellulose under the studied conditions does not follow first-order kinetics. Conversely, the second-order rate constant (*k*_2_) and equilibrium sorption capacity (*q*_e,cal_) were obtained from the linear plot of *t*/*q_t_* vs *t*. The pseudo-second-order model better described the kinetic sorption of MB on quaternary-modified cellulose, as evidenced by a higher correlation coefficient (*R*^2^ = 0.999). Additionally, the calculated sorption capacity (*q*_e,cal_ = 52.36 mg/g) based on the pseudo-second-order model closely matched the experimental value (*q*_e,exp_ = 49.30 mg/g).

The primary method for identifying sorption mechanisms is to fit experimental data to the intraparticle diffusion model (eq 3), which was proposed by Weber and Morris in 1962.^65^ In numerous studies, the *q_t_* vs *t*^0.5^ plot often exhibits multilinearity. As shown in Figure 5d, the sorption data of MB on sulfonated cellulose can be delineated by two straight lines. The initial curved segment of the plot indicates external diffusion (boundary layer diffusion), while the subsequent linear portion represents intraparticle diffusion (diffusion within the polymer network). However, the data points do not intersect with the origin, suggesting that intraparticle diffusion is not the only limiting mechanism. Other factors, such as repulsion between dye molecules and between the sorbent and dye molecules due to the concentration density, also play a significant role. The intercept *C_i_* provides insights into the boundary layer thickness, with a higher intercept indicating a more significant boundary layer effect. The *C_i_* value was found to be 46.74 mg/g. Moreover, the intraparticle diffusion rate constant *k*_i_, derived from the latter portion of the *q_t_* vs *t*^0.5^ plot, was determined to be 0.811 mg/g min^0.5^. Table 1 compares the results of the pseudo-first-order, pseudo-second-order, and intraparticle diffusion kinetic models, including their correlation coefficients, rate constants, and equilibrium sorption capacities.

**Table 1:** Calculated Parameters and Correlation Coefficients of Kinetic Models Determined for the Sulfonated Cellulose for an Initial MB Concentration of 100 mg/L

model	parameter	value
pseudo-first-order	*R*^2^	0.994
	*k*_1_ (1/min)	0.682
	q_e,cal_ (mg/g)	24.12
	q_e,exp_ (mg/g)	49.30
pseudo-second-order	*R*^2^	0.999
	*k*_2_ (g/(mg·min))	0.046
	*q*_e,cal_ (mg/g)	52.36
	*q*_e,exp_ (mg/g)	49.30
intraparticle diffusion	*R*^2^	1.000
	*k*_i_ (mg/g min^0.5^)	0.811
	*C_i_* (mg/g)	46.74

#### Sorption Isotherms

**Figure 6:**
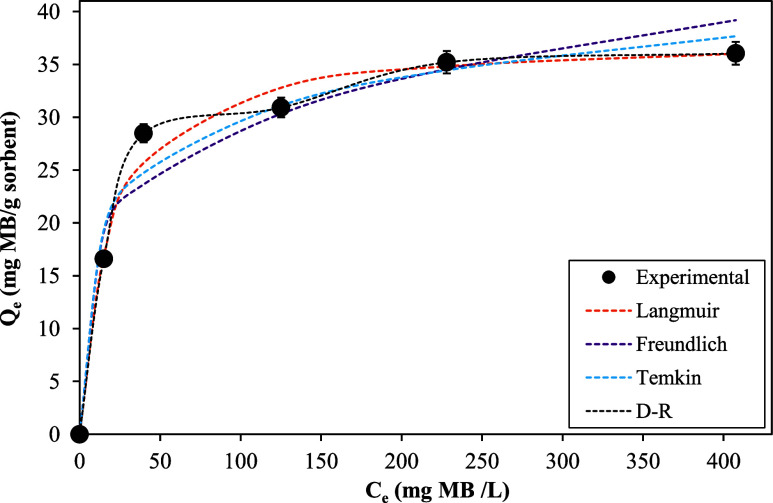
Sorption isotherm plot of MB on sulfonated cellulose with experimental data and theoretical isotherm models.

The sorption isotherms of MB removal by sulfonated cellulose offer a comprehensive understanding of the equilibrium behavior of the sorption process, which was assessed by changing the MB dosage to 50–500 mg/L at a temperature of 25 °C. Figure 6 demonstrates that the sorbent capacity increases with rising initial MB concentration, eventually plateauing at higher concentrations. This can be explained as follows: Ion transport occurs through both conventional and diffusive mechanisms, which are influenced by concentration gradients or, more precisely, by thermodynamic gradients in chemical potential. The activity of an ion is directly proportional to its concentration; therefore, as the concentration of MB increases, so does its activity, leading to an increase in its chemical potential. This rise in chemical potential promotes enhanced diffusion, allowing for greater ion accumulation within the sorbent, which contributes to the observed increase in its capacity.^66^Figure 6 also shows the effect of initial concentration on the MB sorption capacity of sulfonated cellulose experimentally and theoretically via various sorption isotherm models, namely, Langmuir, Freundlich, Temkin, and Dubinin–Radushkevich (D–R), which were employed to meticulously characterize the interaction between MB molecules and sulfonated cellulose. By fitting experimental data to these isotherm models, researchers can extract valuable parameters such as maximum sorption capacity, affinity, and energetics, facilitating a nuanced understanding of the MB removal process by sulfonated cellulose. This detailed analysis aids in optimizing the sorption conditions for efficient and tailored water purification applications.

The Langmuir isotherm assumes monolayer sorption, postulating that MB molecules adhere to identical and independent sorption sites on the sulfonated cellulose surface. It provides insights into the maximum adsorption capacity and the interaction strength between the adsorbate and adsorbent.^67,68^ The Langmuir isotherm equation^69^ is expressed as follows
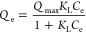
4where *Q*_e_ and *Q*_max_ denote the equilibrium and the maximum sorption capacities (mg/g), respectively. *K*_L_ is the Langmuir isotherm constant (L/mg). *Q*_e_ is calculated as follows in which *V* is the solution volume (L) and *m* is the dry sorbent amount (g)
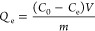
5Conversely, the Freundlich isotherm describes multilayer adsorption on heterogeneous surfaces, accounting for the nonuniform distribution of sorption sites on the sulfonated cellulose matrix. This model is particularly useful for systems where adsorption strength varies across the surface.^70^ The Freundlich isotherm^71^ is given in eq 6
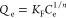
6where *K*_F_ and *n* are Freundlich isotherm constants for adsorption capacity and adsorption intensity of the adsorbent, respectively.

The Temkin isotherm introduces indirect interactions between the adsorbate and adsorbent, considering the effect of adsorbate–adsorbent interactions on the heat of adsorption. This model is applicable when the adsorption process involves some degree of ion exchange between the adsorbate and the surface functional groups of sulfonated cellulose.^72^ The Temkin isotherm model^73^ is described as follows
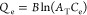
7where *B* = *RT*/*b*_T_, which is the Temkin constant related to the heat of sorption, whereas *A*_T_ (L/mg) is the equilibrium binding constant related to the maximum binding energy. *R* (8.314 J/mol/K) is the universal gas constant, and *T* (K) is the absolute solution temperature.

The Dubinin–Radushkevich isotherm provides insights into the mechanism and energy of adsorption, helping to discern the physical nature of the adsorption process. It is particularly valuable for understanding the pore-filling mechanism and the energy distribution of the adsorption sites on sulfonated cellulose.^74^ It can be expressed as
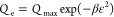
8
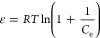
9
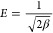
10where β (mol^2^/kJ^2^) is a constant related to the adsorption energy and ε (kJ/mol) is the adsorption potential. Moreover, the parameter β could be used to estimate the mean free energy (*E*), which could distinguish the type of adsorption process. When the magnitude of *E* is less than 8 kJ/mol, the adsorption process is physical adsorption, and when *E* is between 8 and 16 kJ/mol, the process is ion exchange.^75^

Table 2 compiles the isotherm model constants and correlation coefficients, offering a comprehensive insight into the sorption behavior of MB onto sulfonated cellulose and providing a robust foundation for the analysis of the equilibrium relationship between the adsorbate and adsorbent. The Langmuir isotherm revealed a high maximum sorption capacity (*Q*_max_ = 37.65 mg/g) and a relatively low Langmuir constant (*K*_L_ = 0.054 L/mg), indicating strong monolayer sorption with uniform surface coverage. The D–R isotherm provided insights into the sorption energy (β = 0.0027 mol^2^/kJ^2^) and the mean free energy of sorption (*E* = 13.49 kJ/mol), suggesting an ion exchange process with a maximum sorption capacity of 57.90 mg/g. The shape of the Langmuir isotherm exhibits a distinctive “*S*” curve when plotted, representing the saturation of the adsorbent surface as the adsorbate concentration increases. Initially, the sorption capacity increases sharply at low concentrations, as vacant sites on the surface become occupied. As the concentration rises, the sorption rate gradually decreases until reaching a plateau, where the sorption is essentially complete, and further increases in concentration do not lead to additional sorption. This characteristic shape reflects the assumption of a finite number of identical and energetically equivalent sorption sites, forming a monolayer on the adsorbent surface that better describes the behavior of MB sorption on sulfonated cellulose. The sulfonic acid groups are negatively charged, attracting positively charged species (such as cations or dyes like MB). Once these binding sites are filled, no further molecules can bind to the surface, limiting the sorption to a monolayer. Table 3 shows a comparative analysis of various biosorbents for removing MB, focusing on the maximum sorption capacity and equilibrium time under specified initial dye concentrations (*C*_0_). Sulfonated cellulose, investigated in this study, showed a notable sorption capacity of 37.65 mg/g (*C*_0_ = 500 mg/L) with a rapid equilibrium time of 5 min. This indicates that sulfonated cellulose is highly effective in MB removal due to its fast sorption kinetics and significant sorption capacity. According to the reported biosorbents in the literature, citric-acid-modified cellulose exhibits the highest sorption capacity at 211.42 mg/g (*C*_0_ = 200 mg/L) with an equilibrium time of 50 min, making it a strong contender. However, the rapid equilibrium time of sulfonated cellulose provides a distinct advantage in practical applications where time efficiency is crucial. Breadnut peels also performed well with a sorption capacity of 409 mg/g (*C*_0_ = 50 mg/L) and an equilibrium time of 8 min, highlighting their rapid adsorption kinetics. Apple peels achieve a balance with a sorption capacity of 107.52 mg/g (*C*_0_ = 50 mg/L) and an equilibrium time of 100 min. Among agricultural wastes, yellow passion fruit waste and sugar cane bagasse show a close or lower sorption capacity of 44.70 mg/g (*C*_0_ = 28.7 mg/L) and 9.41 mg/g (*C*_0_ = 5 mg/L), respectively, with varying equilibrium times. Sulfonated cellulose stands out due to its superior sorption capacity and rapid equilibrium time combination. Soybean hulls and Terminalia catappa shells also demonstrate significant capacities of 169.90 mg/g (*C*_0_ = 50 mg/L) and 88.62 mg/g (*C*_0_ = 800 mg/L), respectively, with equilibrium times of 180 and 45 min. These results emphasize the effectiveness of various biosorbents, with sulfonated cellulose showing distinct advantages in both capacity and time efficiency.

**Table 2:** Isotherm Model Constants and Correlation Coefficients for the Sorption of MB onto Sulfonated Cellulose

isotherm model	parameters
Langmuir	*Q*_max_ = 37.65 mg/g
	*K*_L_ = 0.054 L/mg
	*R*^2^ = 0.9989
Freundlich	KF = 10.632
	*n* = 4.607
	*R*^2^ = 0.9011
Temkin	*b*_T_ = 445.67 J/mol
	*A*_T_ = 2.15 L/mol
	*R*^2^ = 0.8992
D-R	β = 0.0027 mol^2^/kJ^2^
	*E* = 13.49 kJ/mol
	*Q*_max_ = 57.90 mg/g
	*R*^2^ = 0.8813

**Table 3:** Comparison of Maximum Sorption Capacity and Equilibrium Time of Various Biosorbents for MB Removal

biosorbent	maximum sorption capacity (mg/g)	equilibrium time	references
yellow passion fruit waste	44.70	48 h	(76)
		(*C*_0_ = 28.7 mg/L)	
Tamarind fruit shells	1.72	40 min	(77)
		(*C*_0_ = 20 mg/L)	
Brazil nut shells	7.81	30 min	(78)
		(*C*_0_ = 100 mg/L)	
citric-acid-modified cellulose	211.42	50 min	(79)
		(*C*_0_ = 200 mg/L)	
breadnut peels	409	8 min	(80)
		(*C*_0_ = 50 mg/L)	
apple peels	107.52	100 min	(81)
		(*C*_0_ = 50 mg/L)	
sugar cane bagasse	9.41	3 min	(82)
		(*C*_0_ = 5 mg/L)	
soybean hulls	169.90	180 min	(83)
		(*C*_0_ = 50 mg/L)	
*Terminalia catappa* shells	88.62	45 min	(84)
		(*C*_0_ = 800 mg/L)	
sulfonated cellulose	37.65	5 min	this study
		(*C*_0_ = 100 mg/L)	

#### Sorption Thermodynamics

The sorption thermodynamics of MB onto sulfonated cellulose is a complex and intriguing process that involves the interaction between the dye molecules and the functional groups present in the sulfonated cellulose structure. The sorption behavior is governed by thermodynamic parameters, such as enthalpy (Δ*H*^o^), entropy (Δ*S*^o^), and Gibbs free energy (Δ*G*^o^) changes. The enthalpy change reflects the heat absorbed or released during the sorption process, providing insights into the nature of the interactions between MB and sulfonated cellulose. The entropy change signifies the degree of disorder or randomness in the system, while the Gibbs free energy change determines the spontaneity of the sorption process.^85−88^

The thermodynamic parameters have been calculated using the equations provided below.^89,90^
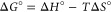
11
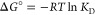
12
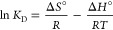
13
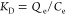
14where Δ*G*° is the change in the Gibbs free energy (kJ/mol), Δ*H*° is the change in the enthalpy (kJ/mol), and Δ*S*° is the change in the entropy (kJ/molK). *K*_D_ is the equilibrium constant (L = g^–1^).

Table 4 encapsulates key thermodynamic parameters, elucidating the sorption of methylene blue onto sulfonated cellulose, providing a detailed analysis of the energy changes and spontaneity associated with the sorption phenomenon. The Gibbs free energy values obtained for MB sorption onto sulfonated cellulose at different temperatures (303, 313, and 323 K) are −8.5, −7.7, and −6.8 kJ/mol, respectively. The negative values of Δ*G*^o^ indicate that the sorption process is spontaneous at each temperature. As the temperature increases, the magnitude of the negative Δ*G*^o^ decreases, suggesting decreasing spontaneity of the sorption at higher temperatures. The enthalpy change (Δ*H*^o^) is −33.5 kJ/mol, indicating the exothermic nature of the sorption process. The negative value of Δ*H*^o^ suggests that the sorption is driven by the release of heat, further confirming the exothermicity. The entropy change (Δ*S*^o^) is −82.6 J/mol/K, indicating a decrease in disorder during the sorption, possibly due to the organization of MB molecules on the sulfonated cellulose surface. Overall, the combination of negative Δ*G*^o^, Δ*H*^o^, and Δ*S*^o^ values implies spontaneous, exothermic, and organized sorption of MB onto sulfonated cellulose, showcasing the potential efficacy of this material as a biosorbent for this dye. The temperature dependence of Δ*G*^o^ highlights the need to consider operating temperatures when designing sorption processes for MB removal using sulfonated cellulose. In terms of large-scale applications, the exothermic nature of the sorption process (negative Δ*H*°) indicates that MB sorption onto sulfonated cellulose releases heat, meaning lower temperatures favor the sorption efficiency. This suggests that the process can be conducted at ambient or lower temperatures without additional heating, reducing energy costs and making it more economically viable. Additionally, the decrease in entropy (negative Δ*S*°) implies increased molecular ordering at the solid–liquid interface as MB molecules are more structured when bound to sulfonated cellulose. This suggests that sorption is highly specific and reliant on well-defined interactions, which may limit sorption in highly complex wastewaters with competing ions. However, this also means that desorption and regeneration processes may require careful optimization to maintain sorption efficiency over multiple cycles.

**Table 4:** Thermodynamic Parameters for MB Sorption onto Sulfonated Cellulose

temperature (K)	Δ**G**° (kJ/mol)	Δ**H**° (kJ/mol)	Δ**S**° (J/mol/K)
303	–8.5	–33.5	–82.6
313	–7.7		
323	–6.8		

#### Desorption of MB and Regeneration of Sulfonated Cellulose

Table 5 presents the regeneration efficiencies of sulfonated cellulose following MB sorption using different regenerating solutions. The provided data indicate the regeneration efficiencies as follows: (1) 12.5 mL 0.2 M HCl + 12.5 mL C_2_H_5_OH (99% efficiency), (2) 12.5 mL 1.0 M HCl + 12.5 mL C_2_H_5_OH (99% efficiency), and (3) 12.5 mL 2.0 M HCl + 12.5 mL C_2_H_5_OH (92% efficiency). The high regeneration efficiencies observed with HCl and ethanol mixtures can be attributed to several factors. Initially, the smaller size and higher concentration of H^+^ from HCl make it effective in initiating the ion exchange reaction with the −SO_3_^–^ groups, facilitating efficient exchange with MB molecules. Additionally, ethanol, an organic solvent with moderate polarity, regenerates the sorbent by disrupting noncovalent interactions. Ethanol interferes with hydrogen bonding between MB molecules and sulfonated cellulose, facilitating the desorption of MB.

**Table 5:** Regeneration Efficacies for MB-Loaded Sulfonated Cellulose with Different Regenerating Solutions

regenerating solution	regeneration efficiency (%)
12.5 mL 0.2 M HCl + 12.5 mL C_2_H_5_OH (d: 0.790 g/mL, ≥99.8%)	99
12.5 mL 1.0 M HCl + 12.5 mL C_2_H_5_OH (d: 0.790 g/mL, ≥99.8%)	99
12.5 mL 2.0 M HCl + 12.5 mL C_2_H_5_OH (d: 0.790 g/mL, ≥99.8%)	92

In summary, the observed regeneration efficiencies suggest that a 1.0 M HCl + C_2_H_5_OH mixture strikes a balance between the desorption capacity and practical considerations, providing an efficient and feasible approach for regenerating sulfonated cellulose after MB sorption.

**Figure 7:**
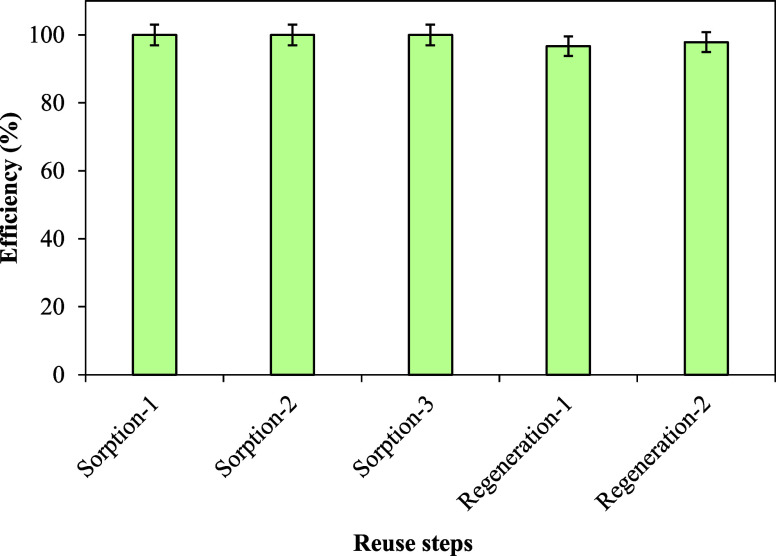
Cycle test of MB sorption by sulfonated cellulose.

The cycle test for the prepared sorbent was conducted to evaluate its reusability and efficiency. Initially, 25 mL of a 5 mg/L methylene blue (MB) solution was treated with 0.05 g of the sorbent for 10 min. The sorbent was then filtered and rinsed with pure water. Subsequently, the MB-loaded sorbent was treated with 25 mL of a regeneration solution (12.5 mL of 0.2 M HCl mixed with 12.5 mL of ethanol) for 30 min. After filtering and washing with deionized water, the sorbent was treated with 0.5 M NaCl solution for another 30 min to convert it back to its Na-form. The sorbent was then rinsed with deionized water and exposed to another 25 mL of a 5 mg/L MB solution. This cycle was repeated 3 times, with the results presented in Figure 7. As depicted in Figure 7, after three cycles, the MB removal efficiency remained above 99%, while the regeneration efficiency was 97 and 98% for the first and second regeneration processes, respectively.

#### Sorption Mechanism

**Figure 8:**
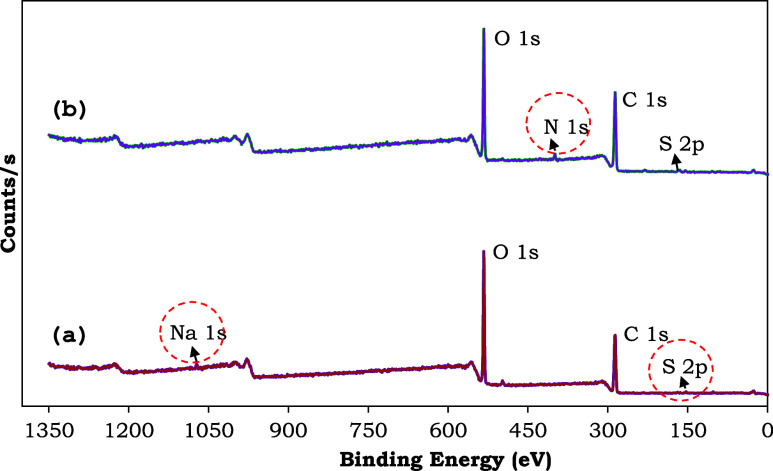
XPS survey of (a) sulfonated cellulose and (b) MB-loaded sulfonated cellulose.

The X-ray photoelectron spectroscopy (XPS) spectrum of sulfonated cellulose before and after MB sorption is shown in Figure 8. As seen in Figure 8, sodium (Na) is present in the sorbent structure prior to the sorption of MB. However, following MB sorption, the Na peak at 1072 eV disappears. This indicates that during the sorption process, an ion exchange reaction occurred between the Na^+^ ions and the MB^+^ dye, as illustrated in eq 15, where C represents the cellulose and MB^+^ is the methylene blue cationic dye.
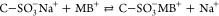
15Furthermore, after the sorption of MB, an N 1s peak formed at about 400 eV, indicating that MB was successfully sorbed onto sulfonated cellulose.

**Figure 9:**
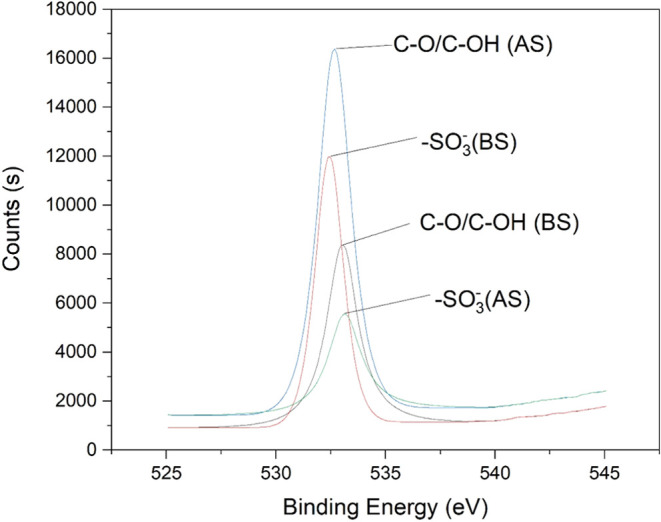
XPS spectrum of the ligands of the O 1s before and after MB sorption (BS: before sorption, AS: after sorption).

An XPS spectrum of O 1s is shown in Figure 9. The peak at 533.0 eV corresponds to C–O/C–OH group^91^ and the peak at 532.4 eV can be attributed to oxygen atoms in −SO_3_ Na groups.^92^ After the sorption of MB, this peak shifted from 532.4 to 533 eV, demonstrating that −SO_3_^–^ groups play a role in MB sorption. This also supports eq 15 and confirms that an ion exchange reaction occurs between −SO_3_Na and MB. After the sorption of MB, the peak at 533.0 eV shifted to 532.7 eV, suggesting that C–OH groups may play a role in MB sorption through hydrogen bonding, thereby enhancing the sorption of MB.

**Figure 10:**
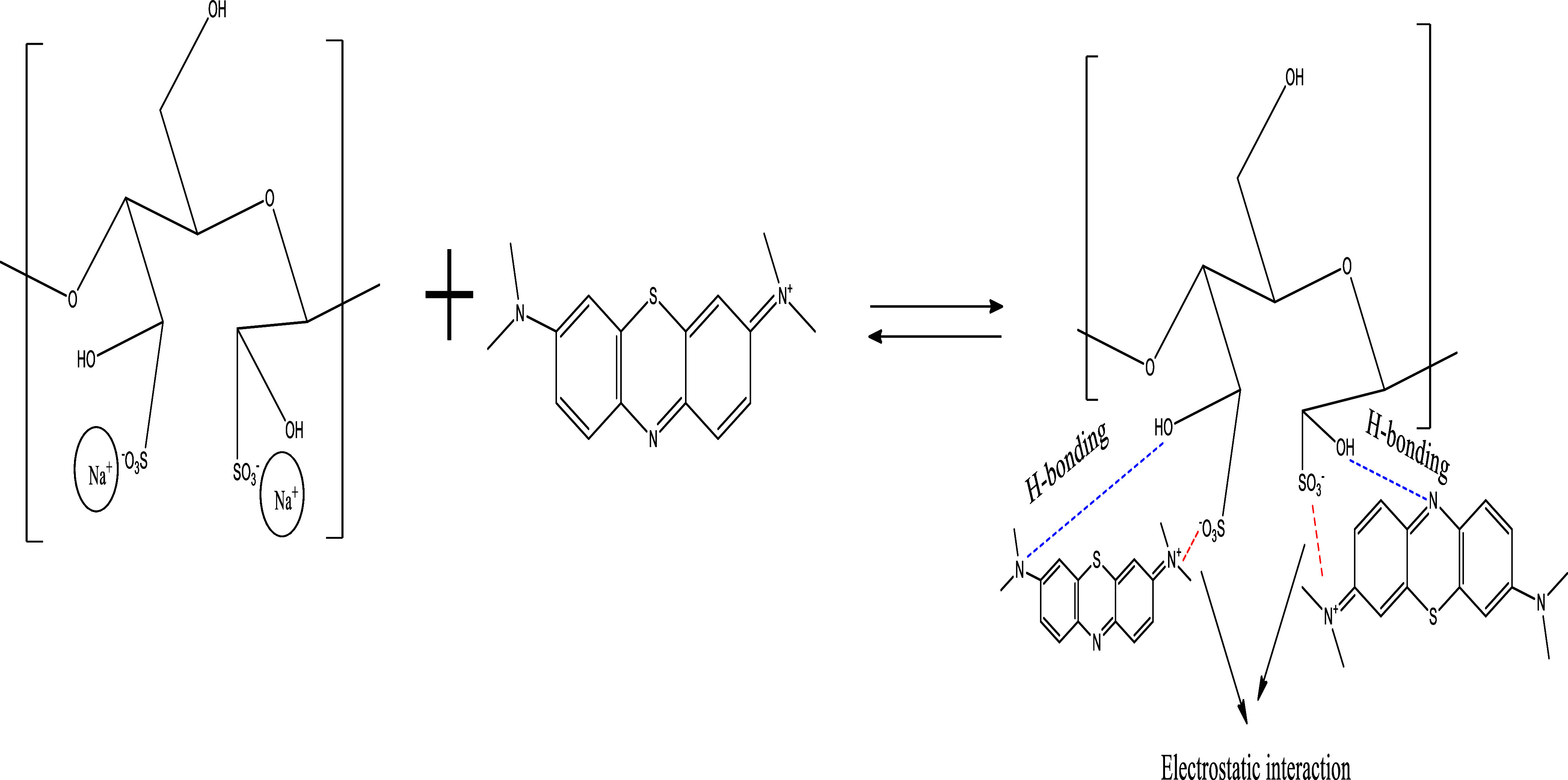
Sorption mechanism of MB by sulfonated cellulose.

Based on these findings, it is concluded that both ion exchange and hydrogen bonding between the C–OH groups of cellulose and the nitrogen atoms in the MB dye contribute to the sorption process. The proposed removal mechanism is shown in Figure 10.

Despite the high sorption efficiency of sulfonated cellulose under controlled conditions, certain limitations must be acknowledged for its application in large-scale wastewater treatment. The sorbent’s capacity may not suffice when addressing wastewater with extremely high dye concentrations. Industrial effluents, often comprising complex mixtures of dyes and competing ions, can lead to diminished sorption performance due to active site saturation and interference effects. Although sulfonated cellulose is both biodegradable and eco-friendly, its capability to sustain high sorption efficiency across multiple cycles is critical. Structural degradation or reduced sorption capacity postregeneration can escalate operational costs, thereby constraining long-term applicability. Furthermore, the intricate composition of industrial wastewater with a blend of organic pollutants, heavy metals, and salts may adversely affect the selectivity and efficiency of methylene blue sorption, presenting additional challenges for practical implementation.

## Conclusions

This study demonstrates the potential of sulfonated cellulose as an efficient and environmentally sustainable biosorbent for the removal of methylene blue (MB) from aqueous solutions. The results revealed rapid sorption, achieving 99% MB removal within 3 min, with the Langmuir model confirming monolayer sorption and a maximum capacity of 37.65 mg/g. Thermodynamic analysis indicated the spontaneity of the sorption process (negative Δ*G*°) and its exothermic nature (Δ*H*° = −33.5 kJ/mol), along with a decrease in system entropy (negative Δ*S*°), suggesting increased molecular ordering at the solid–liquid interface. Regeneration studies confirmed the biosorbent’s reusability, with 0.1 M HCl and ethanol achieving the highest desorption efficiency. The high proton concentration from HCl facilitated ion exchange with −SO_3_^–^ groups, displacing MB molecules, while ethanol disrupted hydrogen bonding, enhancing desorption. Even after three cycles, MB removal remained above 99%, demonstrating an excellent regeneration potential. These findings contribute to the advancement of green chemistry by offering a cost-effective and sustainable solution for dye removal with implications for wastewater treatment and environmental remediation. To enhance the practical application of sulfonated cellulose, further studies should focus on improving its reusability and broaden its applicability to other contaminants. Investigations into efficient regeneration methods, such as chemical desorption, mild thermal treatment, or advanced oxidation processes, could help maintain the sorption capacity over multiple cycles. Modifying the biosorbent through cross-linking or hybrid composites with materials like metal oxides or biobased polymers may improve structural stability and recyclability.

## Experimental Section

### Materials

High-quality chemicals were employed in this study to ensure accurate and reliable results. Sodium metabisulfite (Na_2_S_2_O_5_, 99%), sodium hydroxide (NaOH, 99%), sulfuric acid (H_2_SO_4_, 96%), and nitric acid (HNO_3_, 65%) were procured from Merck. Methylene blue (C_16_H_18_ClN_3_S) with a purity of 99% was supplied from Merck, while sodium periodate (NaIO_4_, 99%) was obtained from Acros Organics. Hydrochloric acid (HCl, 37%) was supplied by PanReac.

### Preparation of Sulfonated Cellulose

**Figure 11:**
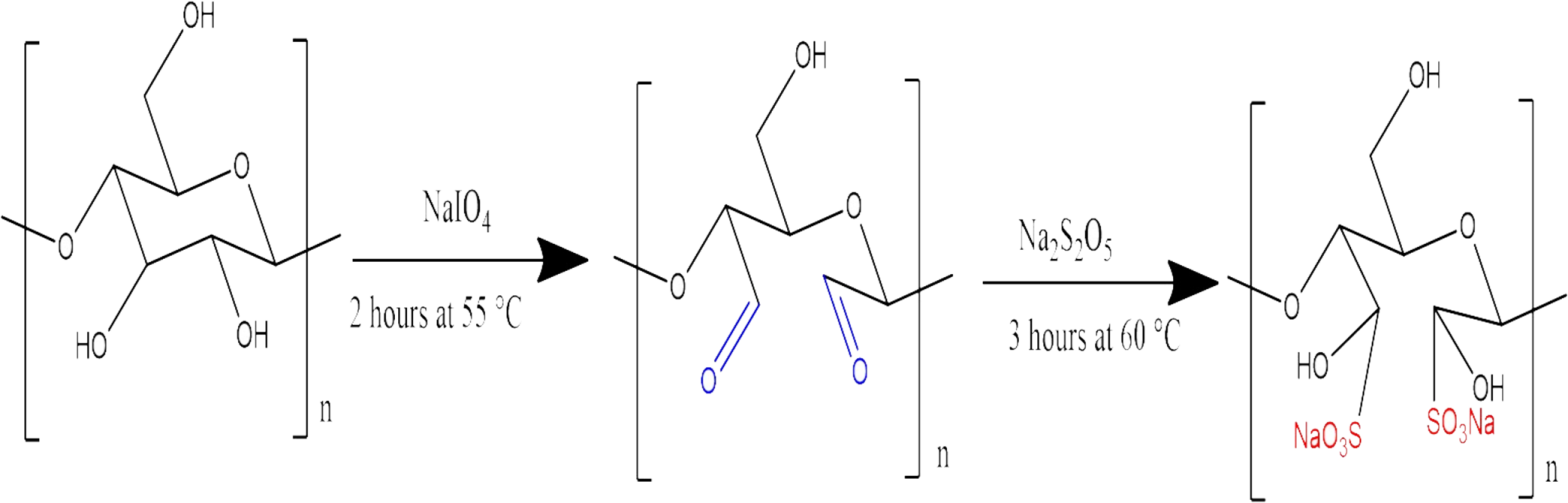
Modification steps of cellulose through sulfonation.

50.0 g of cellulose was oxidized by adding to a 1000 mL solution of 0.038 M NaIO_4_ at 55 °C for 2 h with stirring. The oxidized cellulose was washed with distilled water and then reacted with 1000 mL of 0.242 M Na_2_S_2_O_5_ at 60 °C for 3 h to attach sulfonic acid groups to the cellulose. After this period, the cellulose was filtered, washed, dried in an oven until a constant weight was achieved, and then ground for use in experiments.^61,93,94^ The modification steps of cellulose through sulfonation are shown in Figure 11.

### Characterization of Materials

Utilizing a PerkinElmer Spectrum Two FTIR spectrometer, the changes in untreated and modified cellulose bonding structures were investigated by analyzing IR spectra across the 4000–400 cm^–1^ range.

The Quanta 250 SEM model, a scanning electron microscope, was employed to examine surface properties. Before image acquisition, a thin layer of gold was meticulously applied to the material surfaces, maintaining an accelerating voltage of 3.0 to 5.0 kV.

### MB Removal Studies by Sulfonated Cellulose

First, the prepared sorbent was weighed in specific amounts (0.002, 0.005, 0.05, 0.1, 0.2, and 0.3 g), and then 25 mL of a 5 mg/L MB solution was added to each sample and left to shake for 1 h. Then, the sorbent was separated from the solution by filtration. The remaining MB concentrations in the solution were determined, and removal efficiencies were calculated using eq 16 for each sorbent quantity to determine the optimal sorbent amount.^95^
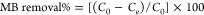
16where *C*_0_ represents the initial concentration of MB (mg/L), while *C*_e_ denotes the equilibrium concentration (mg/L).

To investigate the effect of pH, the determined optimum sorbent amount (0.05 g) was brought into contact with 25 mL of 5 mg/L MB solutions at various pH values (pH: 1.0, 2.0, 3.0, 5.0, 7.0, 9.0) for 1 h.

The kinetic study was performed so that 2.0 g of the sorbent was added to a 1000 mL solution containing 5 mg/L MB, and the mixing process was initiated at 300 rpm. Samples of 10 mL were collected from the solution at specified intervals.

In sorption isotherm studies, 25 mL of MB solutions at concentrations of 50, 100, 200, 300, and 500 mg/L was added to the optimum sorbent at its optimum pH. The mixture was allowed to equilibrate for 1 h at 25 °C.

For thermodynamic studies, 25 mL of a solution containing 300 mg/L MB was added to the optimum sorbent quantity (0.05 g) within the optimal pH range. The mixture was then equilibrated for 1 h in a shaking water bath at 30 °C. The same procedure was repeated at 40 and 50 °C.

Two independent experiments were performed concurrently, and the resulting data were averaged and presented in tables and graphs for analysis. The maximum variation between the two experimental data sets was 3%, and accordingly, a 3% error bar was incorporated into the relevant figures. The concentrations of MB were determined using a UV–visible spectrophotometer (Agilent Carry 60 UV/GB) at λ_max_ of 663 nm.^96^

### Desorption of MB and Regeneration Studies

The regeneration process was performed to recover MB retained by sulfonated cellulose. The regeneration process consisted of two stages. In the first stage, MB was loaded onto the sorbent. 1.0 g of sorbent was weighed, and 100 mL of a 1000 mg/L MB solution, prepared within the optimal pH range, was added. The mixture was shaken for 1 h. After this period, the prepared sorbent was separated by filtration, washed with distilled water, and dried in an oven until a constant weight was achieved. The concentration of MB remaining in the filtrate was measured to calculate the amount of MB retained by the sorbent. In the second stage of the study, recovery of the retained MB from the sorbent was carried out. 0.025 g of MB-loaded dry sorbent was weighed, and 25 mL of HCl and ethanol mixture (1:1, v/v) with different concentrations (0.2, 1.0, and 2.0 M) was shaken for 1 h. Subsequently, the sorbents were separated by filtration. The MB concentration infiltrating into the solution was measured, and the regeneration efficiency for each regenerating solution was calculated according to the following formula^97^
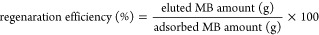
17
